# Supplementary material for: Terahertz nanospectroscopy of plasmon polaritons for the evaluation of doping in quantum devices
Source: Nanophotonics. 2023 Apr 3;12(10):1865–75. doi: 10.1515/nanoph-2023-0064 (PMC11614332; doi:10.1515/nanoph-2023-0064)
Supplement: Supplementary file 1 — Supplementary Material Details [file j_nanoph-2023-0064_suppl_001.docx]

**Supplementary Material: Terahertz nanospectroscopy of plasmon polaritons for the evaluation of doping in quantum devices**

Xiao Guo^1^, Xin He^2,3^, Zachary Degnan^2,3^, Chun-Ching Chiu^2,3^, Bogdan C. Donose^1^, Karl Bertling^1^, Arkady Fedorov^2,3^, Aleksandar D. Rakic^1*^ and Peter Jacobson^2*^

^1*^School of Information Technology and Electrical Engineering,

The University of Queensland, St Lucia, Brisbane, 4072,

Queensland, Australia.

^2*^School of Mathematics and Physics, The University of

Queensland, St Lucia, Brisbane, 4072, Queensland, Australia.

^3^ARC Centre of Excellence for Engineered Quantum Systems, St

Lucia, Brisbane, 4072, Queensland, Australia.

*Corresponding author(s) E-mail(s): a.rakic@uq.edu.au; p.jacobson@uq.edu.au;

Contributing authors: xiao.guo@uq.edu.au; x.he@uq.edu.au; z.degnan@uq.edu.au; chunching.chiu@uq.edu.au; b.donose@uq.edu.au; k.bertling@uq.edu.au; a.fedorov@uq.edu.au; a.rakic@uq.edu.au; p.jacobson@uq.edu.au;

Fig. S1: a typical tip near-field coupling function, ${<q^{2}\exp\left( -2q(H\left( t \right)+b) \right)>}_{t}$, for s-SNOM tip-sample interactions. q is in-plane momenta, H(t)=A(1-cos(Ωt)) is the probe tapping motion during the s-SNOM measurements, b is the distance between effective dipole and the tip apex, A is the probe tapping amplitude, Ω is the probe oscillation fundamental frequency, and t is time. We plot typical examples for tip radius to be 30 nm, 45 nm, and 65 nm.


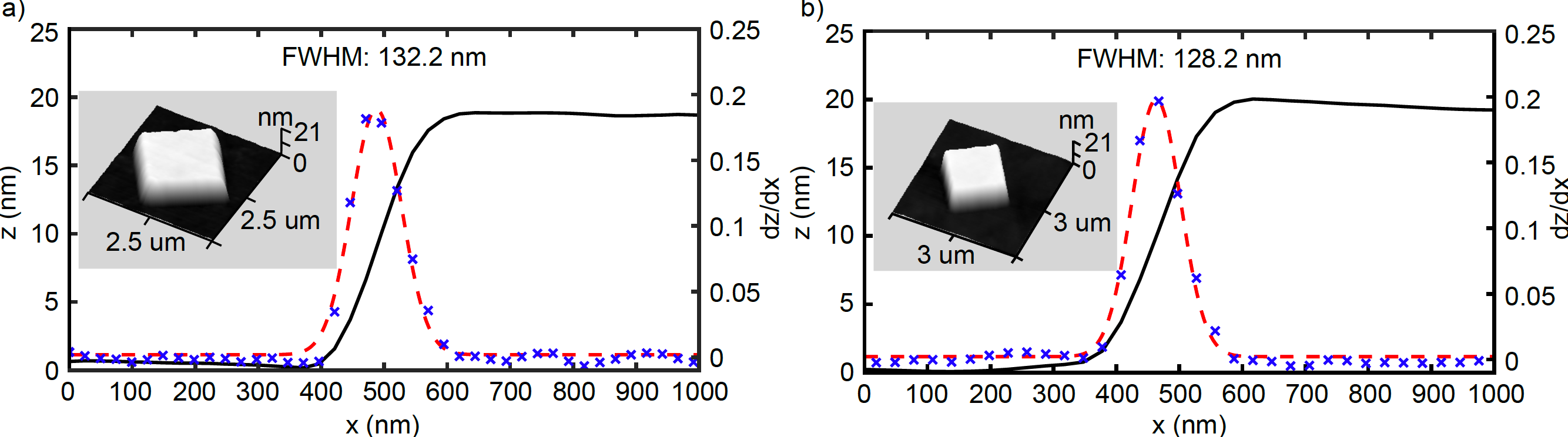


Fig. S2: Line scan across a SiO_2_ square (height variation: 20 +/- 1.5 nm) on Si substrate of commercial AFM calibration sample (TGQ1, TipsNano Co, Estonia) with different probes (25PtIr200B-H, Rocky Mountain Nanotechnology). Insets are the corresponding topography scans. The left axes show the scanning height (z), and the right axes show the spatial gradient of height (dz/dx). A Gaussian kernel is used for fitting to extract the corresponding full-width half maximum (FWHM) to obtain the information of tip radius and spatial resolution.


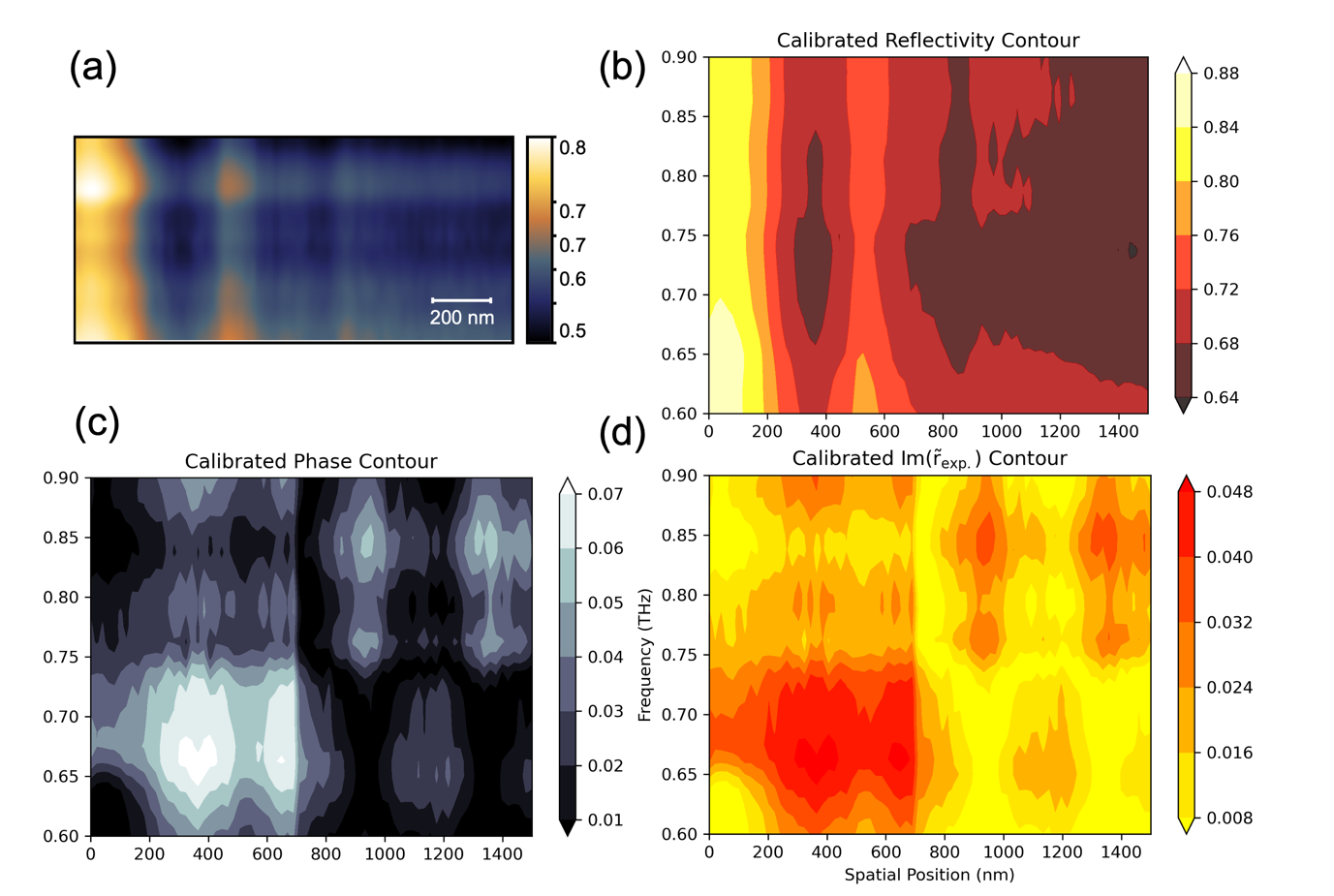


Fig. S3: Calibrated high-resolution (20nm/pixel) hyperspectral data, including **(a)** the amplitude of calibrated reflectivity from hyperspectral data up to 1 THz (with the horizontal axis identical to other three panels), **(b)** the contour of the calibrated reflectivity amplitude, **(c)** the contour of the calibrated reflectivity phase, and **(d)** the contour of the imaginary part of the complex calibrated reflectivity.


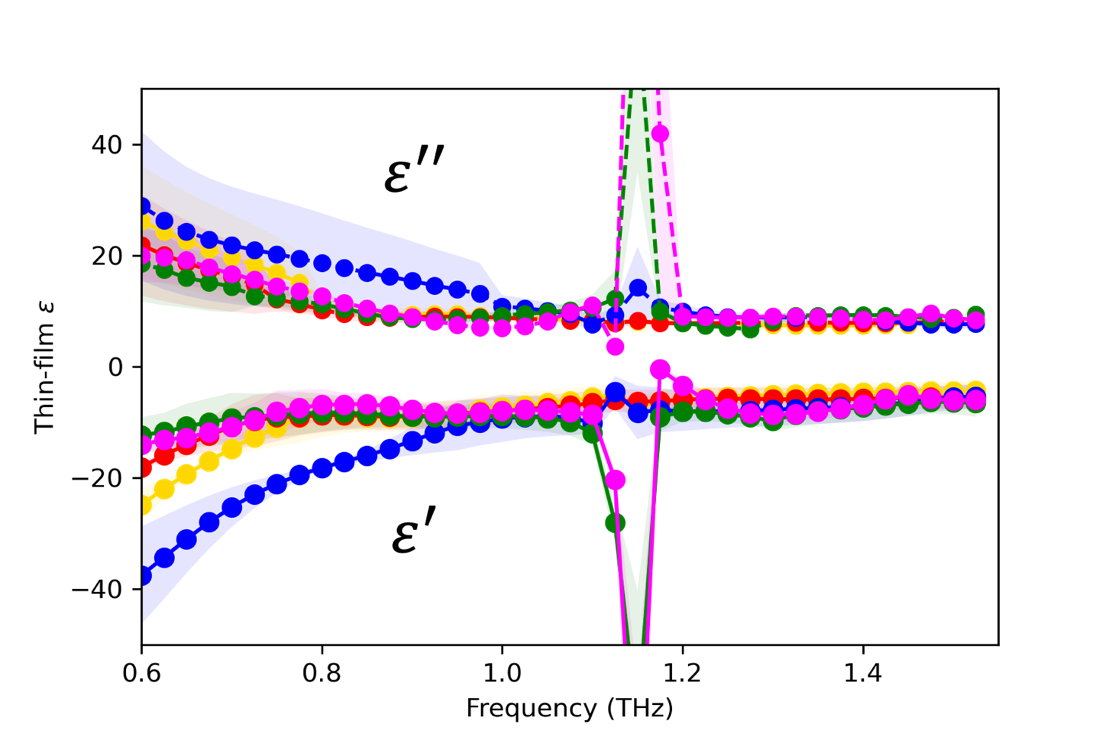


Figure. S4 Extracted complex permittivity of the as-prepared silicon device from 0.6 to 1.5 THz. The peaks around 1.1 THz are due to the existence of strong water vapour line.


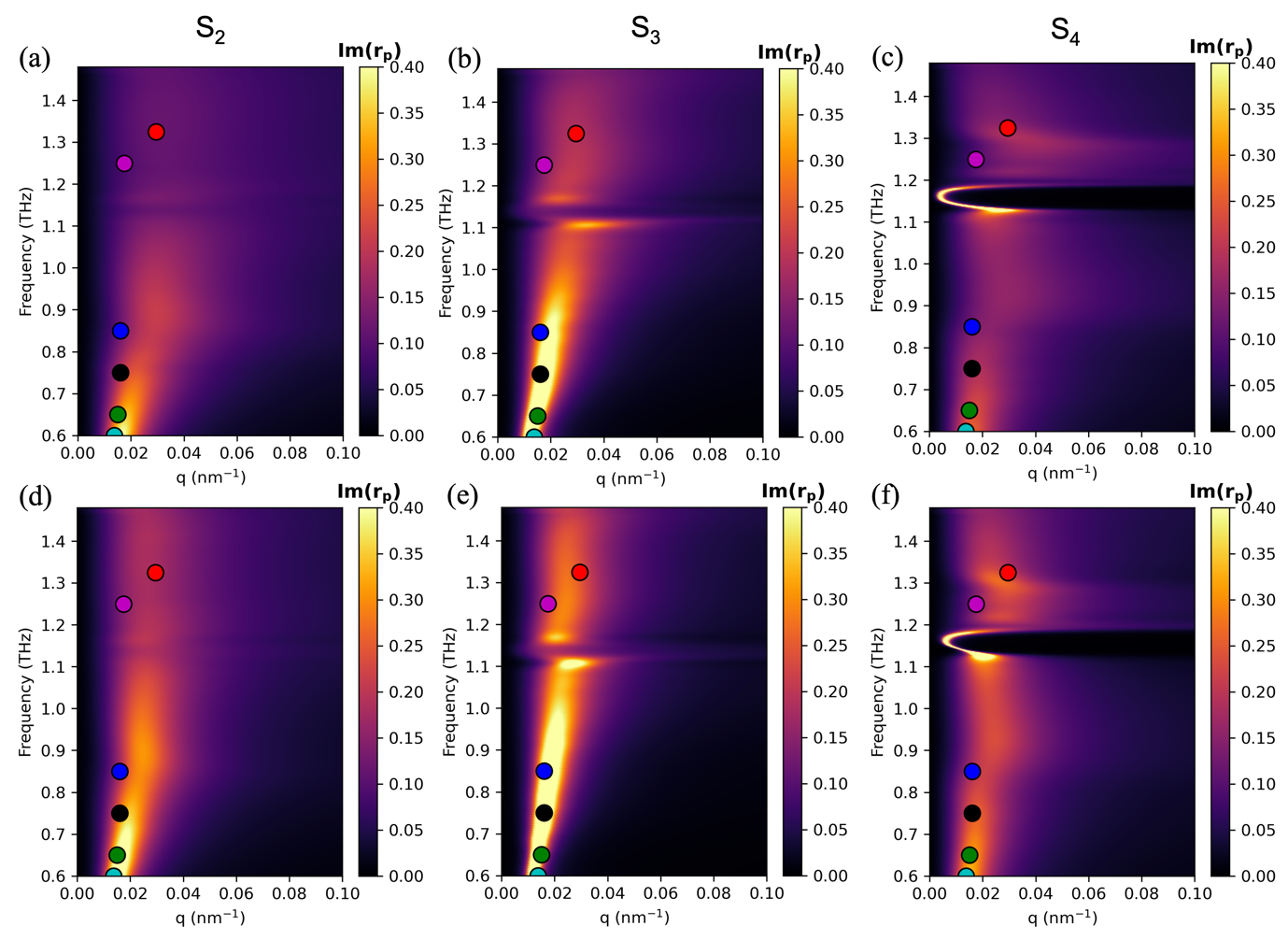


Figure. S5 The measured dispersion curve computed by using the retrieved thin-film thickness (9 nm (a, b, c) and 13 nm (d, e, f) and complex permittivity from the second (S_2_), third (S_3_), and fourth (S_4_) harmonic signals. The circles are extracted in-plane momenta from hyperspectral measurements at the corresponding THz frequencies.


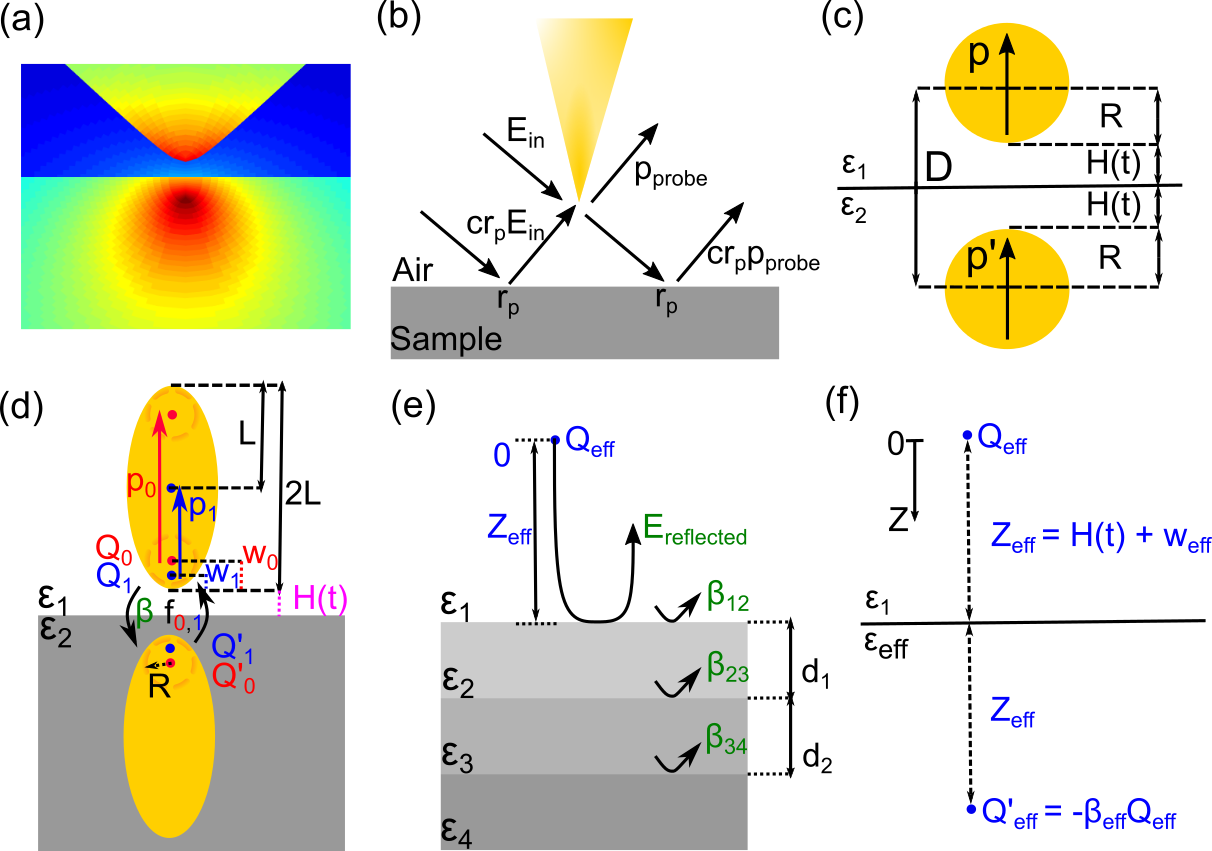


Figure. S6 Dipole effect in s-SNOM: (a) Simulation of the tip-sample's interaction between a 45-nm Au tip on Si substrate ~1 THz to show the s-SNOM near-field interaction. (b) Scattering process occurring in s-SNOM investigations. The s-SNOM tip is hovering at a height $H\left( t \right)$ above the sample with the permittivity $\epsilon$. The incident field $\text{E}_{\text{in}}$ is focused on the tip apex along with a contribution from the sample's surface reflection $cr_{p}\text{E}_{\text{in}}$ due to a wavelength-dependent beam focus (which is larger than the tip radius). The scattering signal is proportional to the overall momentum of the probe and consists of induced momentum $\text{p}_{\text{probe}}$ and $cr_{p}\text{p}_{\text{probe}}$ due to the near-field interaction and surface reflection. $r_{p}$ is transverse magnetic (TM or p) polarised Fresnel coefficient and $c=\exp\left( -i\Delta\phi\right)$ accounts for possible phase retardation between the direct radiation to/from the probe and reflection from the sample (which is usually regarded as 1 in s-SNOM analyses). (c) Point dipole model: the probe is approximated as a dielectric sphere with radius $R$ and effective momentum $p$ due to the near-field interaction. The investigated sample is at a distance $H\left( t \right)$ below the probe and mimicked as a mirror image with momentum $p^{'}$ and the effective tip-probe distance is $D$. (d) Finite dipole model: the probe is approximated as an elongated spheroid (shank length $2L$, radius $R$) with the initial polarisation $p_{0}$ induced by the incident field $E_{\text{in}}$ and the near-field polarisation $p_{1}$ induced by the probe-sample coupling. It assumes only monopoles $Q_{0}$ and $Q_{1}$ near the sample surface participate in the near-field interaction. $W_{0,1}$ denotes the distance between monopole $Q_{0,1}$ and probe apex, $Q_{0}^{'}$ and $Q_{1}^{'}$ are the corresponding mirror images and $H\left( t \right)$ is the realistic tip-sample distance. (e, f) Multilayer treatments on near-field reflections: multiple reflections from the layered system (permittivity $\epsilon_{i}$ and thickness $d_{i}$) are approximated as an effective monopole $Q_{\text{eff}}$ at the distance $Z_{\text{eff}}$ above the surface. $w_{\text{eff}}$is the effective distance between $Q_{\text{eff}}$ and the tip apex.

Fig. S7: THz s-SNOM multilayer extraction of complex-valued optical constant (optical constant: *n*, extinction coefficient: *κ*) and thickness (*d*) for a SiO_2_ square (height variation: 20 +/- 1.5 nm) on a TGQ1 calibration sample: **(a)** a line profile scan across SiO_2_ square and the substrate (see inset for the topography and positions to take the line profile); THz nanospectroscopy is taken at the centre of SiO_2_ square (red circle in the inset); **(b)** Solutions of feasible thickness obtained from multiple harmonics (S_1_ to S_5_) of tip-scattered s-SNOM signals; **(c)** The extracted complex optical constants of SiO_2_ square; Strong water absorption lines above 1 THz are indicated as red arrows; **(d)** Complex-valued optical constants from the 2^nd^ harmonic signal (S_2_) at 20 nm for SiO_2_. Values from s-SNOM measurements are denoted as markers (circle: *n*, triangle: *κ*); Literature values of SiO_2_ measured from far-field THz-TDS systems are shown as lines (solid: *n*, dash: *κ*).
